# Data Quality in Electronic Health Record Research: An Approach for Validation and Quantitative Bias Analysis for Imperfectly Ascertained Health Outcomes Via Diagnostic Codes

**DOI:** 10.1162/99608f92.cbe67e91

**Published:** 2022-04-28

**Authors:** Neal D. Goldstein, Deborah Kahal, Karla Testa, Ed J. Gracely, Igor Burstyn

**Affiliations:** 1 Department of Epidemiology and Biostatistics, Dornsife School of Public Health, Drexel University, Philadelphia, Pennsylvania, United States of America; 2 William J. Holloway Community Program, ChristianaCare, Wilmington, Delaware, United States of America; Sydney Kimmel College of Medicine, Thomas Jefferson University, Philadelphia, Pennsylvania, United States of America; 3 Sydney Kimmel College of Medicine, Thomas Jefferson University, Philadelphia, Pennsylvania, United States of America; Westside Family Healthcare, Wilmington, Delaware, United States of America; 4 Department of Epidemiology and Biostatistics, Dornsife School of Public Health, Drexel University, Philadelphia, Pennsylvania, United States of America; Department of Family, Community, and Preventive Medicine, College of Medicine, Drexel University, Philadelphia, Pennsylvania, United States of America; 5 Department of Epidemiology and Biostatistics, Dornsife School of Public Health, Drexel University, Philadelphia, Pennsylvania, United States of America; Department of Environmental and Occupational Health, Dornsife School of Public Health, Drexel University, Philadelphia, Pennsylvania, United States of America

**Keywords:** electronic health record, data quality, bias, validation, hepatitis C, International Classification of Diseases

## Abstract

It is incumbent upon all researchers who use the electronic health record (EHR), including data scientists, to understand the quality of such data. EHR data may be subject to measurement error or misclassification that have the potential to bias results, unless one applies the available computational techniques specifically created for this problem. In this article, we begin with a discussion of data-quality issues in the EHR focusing on health outcomes. We review the concepts of sensitivity, specificity, positive and negative predictive values, and demonstrate how the imperfect classification of a dichotomous outcome variable can bias an analysis, both in terms of prevalence of the outcome, and relative risk of the outcome under one treatment regime (aka exposure) compared to another. This is then followed by a description of a generalizable approach to probabilistic (quantitative) bias analysis using a combination of regression estimation of the parameters that relate the true and observed data and application of these estimates to adjust the prevalence and relative risk that may have existed if there was no misclassification. We describe bias analysis that accounts for both random and systematic errors and highlight its limitations. We then motivate a case study with the goal of validating the accuracy of a health outcome, chronic infection with hepatitis C virus, derived from a diagnostic code in the EHR. Finally, we demonstrate our approaches on the case study and conclude by summarizing the literature on outcome misclassification and quantitative bias analysis.

## 1. Introduction

Electronic health records (EHRs) are an appealing source of health information for researchers, including data scientists. EHRs capture data recorded during a health encounter, including patient demographics, laboratory orders and results, medical imaging reports, physiologic measurements, medication records, caregiver and procedure notes, and diagnosis and procedural codes (Pollard et al., 2016). The EHR itself can be considered an open cohort representing patients who have engaged with the health care system, or more specifically, the catchment of the EHR (Gianfrancesco & Goldstein, 2021). As such, the EHR contains a depth of information on a breadth of individuals.

In any application of EHR data for secondary analysis, there is a need to understand the quality of the data. After all, EHRs were not originally designed for research. They were intended for medical record keeping, scheduling, and billing purposes (Hersh, 1995). At one extreme, the researcher may treat such data at face value, and assume completeness and accuracy. At the other extreme, the researcher may view the data as wholly unusable, and discard it from analysis completely. Both approaches are far from ideal. Treating data at face value leaves the analysis prone to information bias: either mismeasurement of continuous data or misclassification of categorical data. Treating data as unusable omits potentially vital information from the analysis. This introduces the possibility of information or selection bias when omitted records are systematically different from the retained ones, and at the very least, it needlessly reduces precision of estimates.

Assuming we wish to retain as much data as possible for analysis, accuracy of these data must be determined. Many researchers in the United States use International Classification of Diseases (ICD) codes for ascertaining clinical morbidities. Researchers have found that while presence of a code is a likely indicator of true disease status, the absence of such a code is less reliable for capturing the absence of disease (Goff et al., 2012; Schneeweiss & Avorn, 2005). In other words, specificity of ICD codes is high, while sensitivity is low. This is further compounded by differences in coding standards by clinical specialty (Gianfrancesco et al., 2019), the use of ‘rule out’ diagnostic codes (Burles et al., 2017), as well as the theoretical concern of ‘upcoding,’ or recording wrong diagnoses for the purposes of greater reimbursement (Hoffman & Podgurski, 2013).

The extent and impact of misclassification of a health outcome can be understood through a validation study with accompanying quantitative bias analysis. While these methods are well known in fields like epidemiology, they are nonetheless infrequently used (Hunnicutt et al., 2016; Lash et al., 2014). An instructive summary of common measurement error and misclassification in epidemiology was given in Funk & Landi (2014) and readers are advised to refer to it for a broader overview of the topic as it applies to errors in both outcomes and covariates. We also acknowledge that this problem pervades many fields and there is a rich literature outside of epidemiology and biostatistics worth consulting; for example, see Blackwell et al. (2017) and Schmidt and Hunter (1996).

It is our intention with this article to demonstrate an approach to a validation study and quantitative bias analysis for outcome misclassification assessed via diagnostic codes, motivated from a real-world case study of data derived from the EHR. We seek to connect theory with an applied example and provide a generalizable algorithm for those faced with similar outcome misclassification problems when using EHR data.

## 2. Theoretical Impact of Errors in Diagnosis on Analysis

We turn briefly to theory to show how misclassification may bias estimates. We demonstrate this for both calculation of prevalence and relative risk (RR), where we assume a binary exposure *Z* and misclassification is independent of *Z* (i.e., nondifferential outcome misclassification). We also assume that the outcome is observed as binary *W* (i.e., ICD code present or absent in the EHR) and relates to *X* (true health outcome) by SN and SP. SN and SP do not depend on prevalence of *X* and fully describe misclassification probabilities.

The observed probability (prevalence) of the outcome under the above-specified conditions is *p* = p(W = 1) = rSN + (1-r)(1-SP), where r = p(*X* = 1) is the true prevalence. In other words, the observed prevalence is made up of true cases that are detected plus uninfected individuals who are falsely identified as a case.

Obviously, true and observed prevalence are not guaranteed to be the same, and any analysis that relies on quantifying the number of affected people may be wrong. Standard errors (*SE*) of estimates may also be affected by misclassification because they are equal to (r(1-r)/n)^0.5^ for true and (p(1-p)/n)^0.5^ for the observed, in a sample of size *n*. For example, suppose in a study of *n* = 100 subjects, the perfect diagnostic test is expected to estimate prevalence as *r* = 10% (*SE* 3.0%). However, if an imperfect test with SN = 0.7 and SP = 0.9 was applied, the observed prevalence is expected to be *p* = 16% (*SE* 3.7%). Counterintuitive examples abound, such that in the above scenario, if true prevalence is 25%, then we expect no bias in estimate of prevalence and its standard error because the number of true cases missed exactly equals the number of uninfected falsely classified as infected. The observed and true prevalence are equal when *r* = (1-SP)/(2-SN-SP), leading to a situation where the correct population average rate of diagnosis is obtained even though many wrong people were diagnosed!

If one is interested in how prevalence varies by a group membership, we need to introduce notation for such group or exposure. The observed probability of outcome after treatment *Z* = *i*(*i* = 1 for treated and =0 for untreated) is *p_i_* = p(*W_i_* = 1) = r*_i_*SN + (1-r*_i_*)(1-SP), where r*_i_* = p(*X_i_* = 1), that is, true probability of outcome under the *i^th^* treatment. It follows that observed RR is expected to be RR* = (r_1_SN + (1-r_1_)(1-SP)) / (r_0_SN + (1-r_0_)(1-SP)), which is not always equal to the true RR of r_1_/r_0_ (Green, 1983).

Although it is difficult to intuit the impact of this nondifferential misclassification in a general case, it is clear that RR* tends to be unbiased when SP is nearly perfect, regardless of sensitivity. When *Z* confers no change in risk of *X*, the estimate of RR under nondifferential outcome misclassification is unbiased, but the observed RR* = 1 does not imply true RR = 1. This demonstrates that one is *never* justified in claiming that there is a true proportional increase in risk when none is observed *only* because the outcome is misclassified.

The matters become more complex when SN and SP vary by the exposure, that is, there is differential outcome misclassification with respect to the exposure. Overall, it is not advisable to guess the impact of such misclassification on the bias in the estimate of an exposure’s effect (and even less advisable to predict impact on uncertainty in the estimates and hypothesis tests). There is always a legitimate uncertainty in practice as to whether misclassification is nondifferential, because SN and SP are estimated from validation studies such as ours (typically expensive and therefore small, with nonignorable sampling errors) and not known as constants. Within the range of uncertainty bounds on SN and SP, differential misclassification becomes the most defensible default assumption. Even if data are acquired in a manner that precludes the flow of information between evaluation of outcome and assigned exposure (e.g., done by independent care providers in EHR), differential misclassification can arise by chance alone or due to categorization of truly continuous metrics. A related, albeit distinct, concept, is that of *dependent* misclassification, where multiple variables under study are misclassified, and their probability of being misclassified is dependent upon the correct classification of another variable (Brennan et al., 2021; Lash et al., 2009).

In many real-world problems, estimation of RR is not the final aim, but investigators are rather interested in the burden of a particular disease and how it can be related to treatment, or unevenly distributed among subgroups of people. To answer these types of questions, investigators need to know both RR and prevalence of outcomes. When there is bias in the estimate of RR and the disease is rare, the benefit of treatment in terms of, for example, expected proportion of people cured, can be severely biased (Hsieh, 1991). The bias is potentially even more severe and difficult to anticipate if we are trying to estimate impact of misclassified diagnosis on chance of some distal outcome, such as costly or hazardous treatment or complications of disease: both the effect estimate and prevalence become biased, often leading to substantial undercounting of attributable fractions (Burstyn et al., 2010; Wong et al., 2021).

These matters have been extensively covered in the epidemiology literature for some time (Copeland et al., 1977) yet remain germane to modern analysis and interpretation of EHR data (Desai et al., 2020; Funk & Landi, 2014). In short, the only certain way to not be misled by bias due to misclassification of the diagnosis is to account for it in data analysis, replacing qualitative judgment on bias due to imperfections of data with calculations that capture resultant uncertainty and, ideally, then adjusts for it.

## 3. Generalizable Approach for Describing and Quantifying Outcome Misclassification

**Figure 1.**
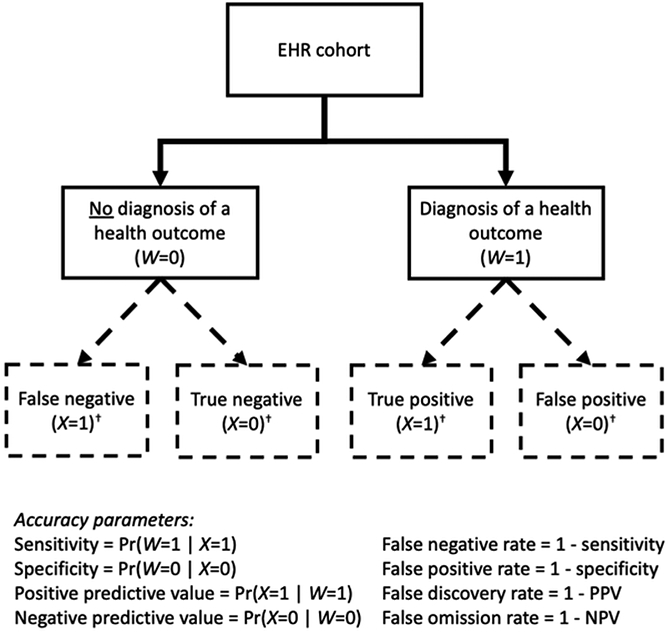
Diagnosis of a health outcome in the electronic health record and its relation to the truth. *‘X’* denotes a patient’s true health outcome while *‘W’* denotes a (potentially incorrect) diagnostic code in the electronic health record. EHR = electronic health record; PPV = positive predictive value; NPV = negative predictive value. ^†^ Dashed lines indicate that the true status is unknown to the researcher.

Continuing with the earlier notation, we let *W* and *X* be the measurements of the error-prone binary EHR-derived diagnosis and perfectly measured true health outcome, respectively. *X* is obtained through validation. In order to proceed, one needs to have an idea of the accuracy of the EHR diagnostic code, which may come from intuition or expert opinion, existing literature, or a de novo validation study. A validation study may occur internally, on a subset of the overall patient sample, or externally, from a different set of patients altogether, provided they are exchangeable with the clinical data under analysis. Sometimes, validation studies arise naturally and only need to be recognized within existing data, as is indeed the case in our illustrative case study presented in sections 4-6. Figure 1 depicts the situation where *W* is known in the EHR, but *X* is not.

To arrive at the needed accuracy parameters (i.e., PPV, NPV, SN, SP, and their complements) one could conduct a validation study or identify if a subcohort of individuals already exist in the EHR where *X* is known for both cases of *W* (=0 or 1). Provided the subcohort is exchangeable with the full cohort, we can estimate these accuracy parameters via logistic regression (Cai et al., 2015). The estimates of PPV, NPV, false omission rate (FOR; the complement of NPV), and false discovery rate (FDR; the complement of PPV) are obtained from:

(1) $$\log\text{it}(X)=\beta_{0}+\beta_{1}W$$

where *β*_0_ and *β*_1_ are parameters, and *X* and *W* are true (measured only in validation study) and observed (on everyone) binary variables, respectively. To estimate PPV, we expit(*β*_0_* + *β*_1_*) and to estimate FOR, we expit(*β*_0_*), where expit(*β*) = exp(*β*) / [1 + exp (*β*)]. NPV is 1 – FOR and FDR = 1 – PPV. Superscripts * denote estimates obtained from regression. Precision is estimated by bootstrapping and is conventionally expressed as 95% confidence interval (CI), although more direct options are available in some statistical platforms, such as maximum likelihood estimation (MLE) implemented in PROC LOGISTIC in SAS (Cary, NC). For sparse data, one can substitute exact logistic regression (Wilson & Lorenz, 2015) or Firth’s logistic regression (Puhr et al., 2017), but bootstrapping is a sensible default approach. The estimates of SN, SP, false positive rate (FPR; the complement of SP), and false negative rate (FNR; the complement of SN) are obtained by swapping the regressors in Equation 1:

(2) $$\log\text{it}(W)=\alpha_{0}+\alpha_{1}X$$

where α_0_ and α_1_ are parameters, and *X* and *W* are true and observed binary variables, respectively. To estimate SN, we expit(α_0_* + α_1_*) and to estimate FPR, we expit(α_0_*). SP = 1 – FPR and FNR = 1 – SN.

To consider misclassification differential with respect to a covariate *Z*, the validation logistic model includes parameters specific to each combination of *W* and *Z*. For example, for binary *Z*, we may construct the following validation logistic regression model of (*X*∣ *W,Z*):

(3) $$\log\text{it}(X)=\beta_{00}+\beta_{10}W+\beta_{01}Z+\beta_{11}W\times Z$$

leading to the following estimates of the four required predictive values (for strata defined by the value of exposure *Z* in the second digit of the subscript): FOR_0_ = expit(*β*_00_*), FOR_1_ = expit(*β*_00_* + *β*_01_*), PPV_0_ = expit(*β*_00_* + *β*_10_*), PPV_1_ = expit(*β*_00_* + *β*_10_* + *β*_01_* + *β*_11_*). NPV = 1 – FOR and FDR = 1 – PPV. Again, by swapping *X* and *W* regressors, one can arrive at estimates of SN and SP:

(4) $$\log\text{it}(W)=\alpha_{00}+\alpha_{10}X+\alpha_{01}Z+\alpha_{11}X\times Z$$

leading to estimates (for strata defined by the value of exposure *Z* in the second digit of the subscript): SP_0_ = expit(α_00_*)−1, SP_1_ = expit(α_00_* + α_01_*)−1, SN_0_ = expit(α_00_* + α_10_*), and SN_1_ = expit(α_00_* + α_10_* + α_01_* + α_11_*).

Extension to more covariates beyond *Z* is trivial albeit tedious, placing ever-increasing demands on the validation data to be informative of the strata-specific effects, while at the same time requiring sufficient sample size. Others have described this problem and supplied a solution in the presence of validation data with diagnosis used as predictor variable (Tang et al., 2015). The advantage of the presented approach is that the equality across strata can be tested and a parsimonious model selected using standard regression techniques. This can help focus efforts to improve the quality of data captured in the EHR in subpopulations where the issue may be more acute. We present the logistic form of the validation model but any technique that predicts probabilities should be suitable, for example, probit or log-binomial regressions.

It is also worth mentioning that there are other methods readers may be familiar with for calculating the parameters needed for a bias analysis from the validation sub-study. For example, the classical 2 x 2 table can be used to cross tabulate the imperfect and perfect binary health outcome indicators, *W* and *X*, respectively (see Tables 2 and 3, for example). We have presented but one approach that conveys certain advantages: it is easier to setup computationally and is more flexible, both to the operationalizing of *W* and *X*, as well as adding strata of *Z*.

If a validation study is unavailable then one must turn to the literature or expert opinion in order to inform the validation parameters, for nondifferential outcome misclassification this would include SN and SP (or PPV and NPV) and for differential outcome misclassification this would include SN and SP estimated at the levels of the exposure (same for PPV and NPV). To proceed with our approach would require operationalizing the values as distributions based on a logit transformation. Researchers who face such a situation, or are newer to bias analysis, are advised to start by applying methods detailed in Lash et al. (2009).

### 3.1. Probabilistic Bias Analysis of Outcome Misclassification With and Without the Presence of an Exposure

A probabilistic (quantitative) bias analysis seeks to assess the sensitivity of results due to systematic errors in a study, while also capturing random errors, both in terms of the magnitude and directionality of estimates (Gustafson & McCandless, 2010; Lash et al., 2009; MacLehose & Gustafson, 2012; Phillips & LaPole, 2003). The following is an overview of our approach to identifying and quantifying outcome misclassification using probabilistic bias analysis of a study aiming to estimate true prevalence of the outcome. A probabilistic bias analysis of outcome misclassification on prevalence of *X* would proceed as follows:

1. Estimate coefficients and standard errors of *β*_0_ and *β*_1_ through application of Equation 1, detailed above, in the validation model.
2. Calculate $\pi =p(X=1|W)=\text{expit}({\dot{\beta }}_{0}+{\dot{\beta }}_{1}W)$, using the imperfect classifier, *W*, in the main study for each person in the cohort who does not have *X* measured, where ${\dot{\beta }}_{0}$ and ${\dot{\beta }}_{1}$ are each sampled from a normal distribution with the means and variances of *β*_0_* and *β*_1_*, respectively.
3. Simulate potential values of $\dot{X}$ from Bernoulli(*π*).
4. Repeat steps 2–3 many times to obtain a distribution of $\dot{X}$ values that reflect simulated true values that would have been observed if there was no misclassification, informing what values Pr(*X*) may take given our data and models. The superscript ‘dot’ stresses that these are simulated values of *X*, not actual true values. As such, this is not a true misclassification adjustment, but rather a sensitivity analysis covering plausible scenarios. Not all simulated values of $\dot{X}$ are equally plausible given data and models, but probabilistic bias analysis does not take this into account (MacLehose & Gustafson, 2012).

This approach can readily be extended to account for an additional covariate, exposure *Z*, for purposes of estimating a RR (or odds ratio), though it must be noted that conditioning of misclassification on more than one covariate appears to be rarely described (though routinely considered in statistics). The extension of the above algorithm to differential outcome misclassification on RR of *X* due to binary exposure *Z*, would proceed through the following steps:

1. Estimate coefficients and standard errors of *β*_00_, *β*_10_, *β*_01_, and *β*_11_ through application of Equation 3, detailed above, in the validation model.
2. Calculate $\pi =p(X=1|W,\;Z)=\text{expit}({\dot{\beta }}_{0}+{\dot{\beta }}_{10}W+{\dot{\beta }}_{01}Z+{\dot{\beta }}_{11}W\times Z)$ using the imperfect classifier, *W,* and exposure, *Z*, in the main study for each person in the cohort without measurement of *X*, where ${\dot{\beta }}_{0}$, ${\dot{\beta }}_{10}$, ${\dot{\beta }}_{01}$, and ${\dot{\beta }}_{11}$, and are each sampled from a normal distributions with the mean and variances of *β*_00_*, *β*__10__*, *β*_01_*, and *β*_11_*, respectively.
3. Simulate potential values of $\dot{X}$ from Bernoulli(*π*).
4. Estimate $\overset{\dot{}}{RR}$ relating *Z* to $\dot{X}$ in the main study that lacks *X*. The resulting $\overset{\dot{}}{RR}$ reflects what RR can be due to misclassification, given data and models. We estimate $\overset{\dot{}}{RR}$ via Poisson regression with robust standard errors (Zou, 2004), appropriate for a cohort design; for case-control sampling, one may also estimate the odds ratio through logistic regression at this step.
5. To account for random errors in estimation of RR, we sample $\log(\overset{\ddot{}}{RR})$ from distribution of normal($\log(\overset{\dot{}}{RR})$, $\text{var}(\overset{\dot{}}{RR})$).
6. Repeat steps 2–5 many times to obtain a distribution of $\overset{\ddot{}}{RR}$ that reflects possible values of what would have been observed in absence of misclassification, given our data and models. We again note that not all simulated values are equally plausible given data and models, but that is not considered in a probabilistic bias analysis.

One approach to account for the situation where implausible estimates arise during the bias analysis simulation would involve weighting parameter estimates of interest ($\overset{\ddot{}}{RR}$ in our example) by likelihoods of models that they are derived from, which is akin to likelihood weighting (Russell & Norvig, 2003, p. 514). We demonstrate this in our case study in section 6, although we emphasize that this is a stopgap measure for a general problem of probabilistic bias analysis as practiced in health research: lack of mechanism to account for ‘poor’ simulation realizations. The only solution that has been offered is to discard simulation realizations that are incompatible with data, for example, leading to undefined effect estimates such as negative odds ratios (Lash et al., 2009). However, discarding all undefined estimates does not offer a complete solution reflective of the reality of complex data and models if it unreasonably treats all remaining simulation realizations as equally likely. Gustafson & MacLehose (2012) propose to bootstrap distribution of retained simulation realizations, while Stayner et al. (2007) utilize weighting by partial likelihood. Yet all of this falls short of consideration of full likelihood, starting with plausibility of simulated misclassification parameters and this would ultimately lead to fully Bayesian approach, not probabilistic (Monte Carlo) bias (sensitivity) analysis. This would be an appropriate next step in either refining our method or leading down a different path of adapting existing Bayesian methods.

Further, there is a rich statistical literature on how to approach the general problem of bias analysis and we seek here to merely illustrate the idea and implementation behind one of the simplest ones, acknowledging that it does not adjust for bias, but rather provides an idea of its systematic impact while also capturing random errors (Jurek et al., 2013; Lyles et al., 2011). For recent guidelines on meeting analytical challenges of error-in-exposure, for example, when diagnosis is used to predict a future event, the reader is referred to these articles (Keogh et al., 2020; Shaw et al. 2020). All such approaches require information on SN and SP that can be derived from modeling *W* as a function of *X* and *Z*, as described earlier. Other methods exist that involve evaluation of likelihood functions associated with each ‘imputation’ (Edwards et al., 2013; Högg et al., 2017).

## 4. Case Study of a Misclassified Diagnostic Code in the EHR

Chronic hepatitis C virus infection (HCV) causes considerable morbidity and mortality in the United States and, as of 2015, was estimated to affect 2–4 million people nationally (Ly et al., 2016; Polaris Observatory HCV Collaborators, 2017). Groups particularly at risk for infection include the ‘baby boomer’ birth cohort (1946–1964), people who inject drugs, institutionalized individuals, and those who are homeless, undocumented, or incarcerated (Denniston et al., 2014). With the recent widespread introduction of direct acting antivirals, the ability to treat and cure HCV is markedly improved over interferon-based regimens that were less effective, had a worse side-effect profile, and required longer therapy (Manns et al., 2006). Further, as restrictions surrounding HCV therapy continue to be further lifted, including the ability of nonspecialists to prescribe it and lack of a urine drug screen test requirement, combined with the 2020 recommendation for one-time screening among adults, many additional patients will become eligible for treatment (Breskin et al., 2019; Marshal et al., 2018; US Preventive Services Task Force [USPSTF] et al., 2020). Thus, there is now a justification to reengage patients to confirm and treat HCV infection.

As data scientists, we may be engaged in a variety of research aims pertinent to HCV, and at our disposal are data abstracted from the EHR. For example, the health care center may wish to know the prevalence of HCV among their patients for the purposes of allocating resources for testing and treatment. Such an analysis may also be useful for the health department in order to ascertain community prevalence of HCV based upon the catchment of the center. Or perhaps the health care center would like to know for a given patient, what is the likelihood of that individual having HCV based on presence or absence of a corresponding diagnosis in the EHR: the positive and negative predictive values of the ICD codes, respectively. Finally, perhaps the clinic would like to know how likely is it that a certain exposure is associated with a diagnosis of HCV for purposes of intervening on the exposure.

As detailed in section 1, the use of an ICD code to ascertain accurate HCV status may be subject to misclassification, including both false negatives—a missing code—or false positives—an inaccurate code. First, patient self-report may have been the reason for documentation in the EHR. Results from the National Health and Nutrition Examination Survey 2001–2008 indicate a general lack of awareness and suboptimal knowledge of HCV infection in the United States (Denniston et al., 2012). Second, documentation may have occurred due to a positive screening, as opposed to a positive confirmatory test. A positive screening test (i.e., reactive HCV antibody) indicates past *or* present infection; it does not prove active HCV, which requires presence of viremia as detected by polymerase chain reaction or viral load assays. Relatedly patients may have spontaneously cleared the virus (Aisyah et al., 2018). Third, the diagnostic code may have been recorded to rule out HCV contingent on further testing, and fourth, a diagnosis may have been recorded by the clinician in free-text notes but never appeared as an ICD code. Taken together, there are multiple reasons why it may be dubious to rely on the EHR ICD code alone to identify those with HCV.

We have several options with how to proceed. First, we may conduct the analysis naïvely, and use the data at face value. Alternatively, we may recognize the limitations of the data and perform quantitative bias analysis to determine the impact of the misclassification. This can be as straightforward as using simple algebra to correct the observed measures or employing more sophisticated sensitivity analyses or simulations to describe plausible ranges of the effect estimates (Funk & Landi, 2014; Gustafson, 2004).

## 5. Description of a Validation Approach for the HCV Diagnostic Code

Our case study employed two data collection periods at an urban federally qualified health center (FQHC). First, we assembled a cohort of adult patients ≥18 years of age seen between November 1, 2016, and October 31, 2018. This time period corresponded with the FQHC’s definition of “active” patients seen ≥1 time in the past 2 years and predates a change in the HCV testing policy. During this time, the FQHC engaged in risk-based screening for HCV, based on either known or disclosed risk factors, or symptomology. Hereafter this cohort is referred to as the ‘risk-based cohort.’ The apparent (observed) presence or absence of HCV was determined by abstracting the following ICD-10-CM codes from the EHR: B18.2 (Chronic viral hepatitis C), B19.20 (Unspecified viral hepatitis C without hepatic coma), B19.21 (Unspecified viral hepatitis C with hepatic coma), and B19.2 (Unspecified viral hepatitis C). These codes were chosen a priori based on the coding practice of the FQHC and were believed at the time to capture the preponderance of cases, albeit imperfectly.

In the second data collection period, for purposes of validating the diagnostic code, we assembled a cohort of patients seen at the FQHC from January 1, 2019, through July 31, 2019, for whom there was no EHR-recorded diagnosis of HCV as per ICD-10-CM codes listed above. In the 2 months prior to formation of this second cohort, universal HCV screening was implemented for all adult patients ≥18 years of age. The universal screening laboratory test is an anti-HCV antibody assay that, if reactive, reflexively checks for detectable HCV RNA, where a double positive result indicates chronic HCV infection (Centers for Disease Control and Prevention, 2013). This second cohort provides insight into the prevalence of HCV *without a known or disclosed risk factor*, and hereafter will be referred to as the ‘universal cohort.’

We aimed to determine validity of documentation of HCV in the EHR in the risk-based cohort. We emphasize this is not accuracy of testing, but rather the accuracy of diagnostic code within the EHR. To validate true HCV infection (denoted as *X* = 1 when confirmed and *X* = 0 otherwise) when an HCV diagnostic code was recorded in the EHR (the potentially misclassified variable, denoted as *W* = 1 when present and *W* = 0 otherwise), we first undertook a manual chart review utilizing data from both discrete and nondiscrete fields. Discrete data were derived from laboratory reports, including results from any HCV screening test (antibody present) and results from any HCV confirmatory test (virus present). Nondiscrete data were obtained from free-text encounter notes and may have additionally indicated provider documentation of test results, HCV treatment and possibly cure, HCV risk factors (e.g., incarceration, injection drug use, sexual minority male), or patient self-report of HCV Second, starting in December 2018, outreach was conducted among patients suspected of having HCV (*W* = 1). This outreach attempted to reengage patients with care and corroborate suspected diagnosis through laboratory confirmation.

**Figure 2.**
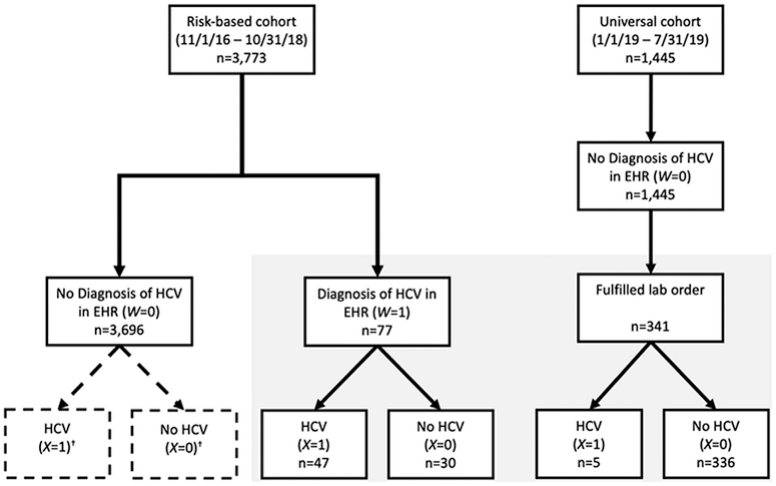
Study enrollment and decision steps to classify an individual’s chronic hepatitis C virus infection status from the electronic health record among patients seen at an urban federally qualified health center. *‘X’* denotes a patient’s true infection status, while *‘W’* denotes a (potentially incorrect) diagnostic code in the electronic health record. Gray shaded box depicts the validation subcohort (*n* = 418). HCV = chronic hepatitis C virus infection; HER = electronic health record. ^†^ Dashed lines indicate missing HCV status for patients without laboratory testing in risk-based cohort.

Given that patients in the risk-based cohort received this additional scrutiny to confirm true HCV infection when a risk factor was identified—and thus documented in the EHR—we could calculate Pr(*X* = 1∣*W* = 1), or the positive predictive value (PPV) of an HCV diagnostic code indicating true infection. However, we did not know true infection status (*X*) when an HCV diagnostic code was absent from the EHR (*W* = 0) in this cohort. Hence, we were unable to calculate Pr(*X* = 0∣*W* = 0), or the negative predictive value (NPV) of lack of an HCV diagnostic code indicating no true infection and Pr(*X* = 1∣*W* = 0) or 1-NPV of lack of an HCV diagnostic code indicating true infection. To allow these calculations, as well as overall prevalence, Pr(*X* = 1), we assumed that the two cohorts were exchangeable (an assumption that is discussed later) and the *W* = 0 patients from the universal cohort were pooled with the *W* = 1 patients from the risk-factor-based cohort to form a validation subcohort. Figure 2 depicts the study enrollment process into the respective cohorts.

Whereas PPV and NPV are dependent upon Pr(*X* = 1) and therefore may be difficult to generalize to other settings with a differing prevalence of HCV, we also calculate sensitivity (SN) and specificity (SP) from the validation cohort. The accuracy of an EHR diagnosis for HCV (*W* = 1) given true HCV (*X* = 1) can be written as SN = Pr(*W* = 1∣*X* = 1) and the absence of an EHR diagnosis for HCV (*W* = 0) when there is no true HCV (*X* = 0) can be written as SP = Pr(*W* = 0∣*X* = 0).

The risk-based cohort included 3,773 patients with characteristics provided in Table 1. The majority of patients were female (67%), non-White (69%), non-Hispanic (75%), and did not have private insurance (68%). An ICD code corresponding to HCV was recorded in 77 patients (2%), among whom chart review, combined with outreach, identified 47 patients (1% of total cohort; 61% of those with a diagnosis) as having confirmed HCV. The universal cohort included 1,445 patients without known risk factors, of whom 341 (24%) had a result of an HCV test: 5 (2%) were identified as having chronic HCV through a positive confirmatory test. There were no qualitative differences between the risk-based and universal cohorts in the characteristics examined, nor were there any qualitative differences between those with a resulted lab test and those without a resulted lab test in the universal cohort (Table 1), thus supporting our decision to combine a subset of individuals into a single validation subcohort. The validation subcohort consisted of 418 patients (*n* = 77 from the risk-based cohort plus *n* = 341 from the universal cohort who fulfilled the lab order).

## 6. Application of the Model to the Case Study

### 6.1. Nondifferential Outcome Misclassification

Working through Equations 1 and 2 and applying bootstrap yields, the following estimates of the accuracy of our classifier in the subcohort were obtained: PPV = 61% (95% CI: 51%, 73%), NPV = 99% (95% CI: 97%, 100%), SN = 90% (95% CI: 82%, 98%), and SP = 92% (95% CI: 89%, 95%). For comparison, we also present in Table 2 the simple cross-tabulation of the validation subcohort. Based on this 2 x 2 table, the accuracy of the EHR diagnosis of HCV in the sub-cohort was: PPV = 61% (95% CI: 49%, 72%), NPV = 99% (95% CI: 97%, 100%), SN = 90% (95% CI: 79%, 97%), and SP = 92% (95% CI: 89%, 94%). As expected, the point estimates and CIs in the two approaches agree.

With these validation parameters obtained from the validation subcohort, and our goal to estimate the true prevalence of HCV in the risk-based cohort, we apply the algorithm from section 3.1 to construct the model of true prevalence based on the observed HCV EHR diagnosis in the risk-based cohort. For demonstration purposes, we simulated 1,000 potential values of $\dot{X}$ for each individual, obtained the sample mean for each iteration as an estimate of true prevalence, and calculated the 2.5, 50, and 97.5 percentiles of the resultant $\dot{X}$ distribution over the 1,000 simulations.

**Figure 3.**
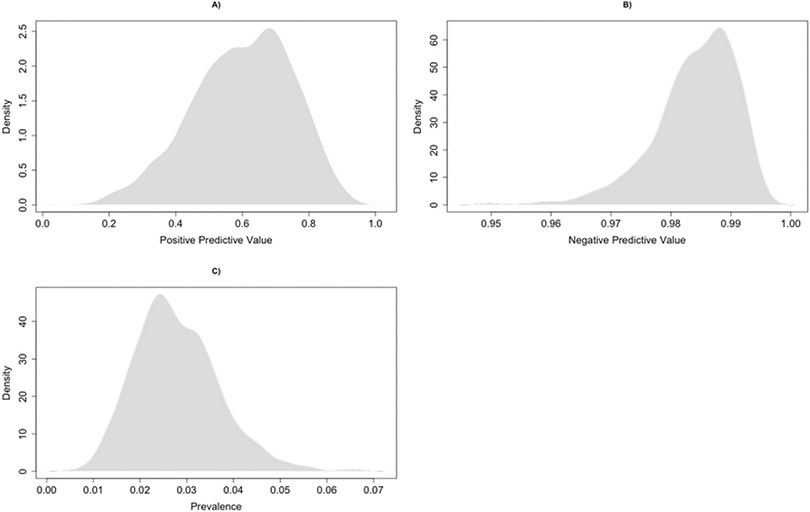
Density plots depicting the distribution of simulated positive predictive value (A), negative predictive value (B), and true prevalence (C) obtained during the quantitative bias analysis of outcome misclassification.

The distribution of the key parameters realized during this simulation are shown in Figure 3. The median (2.5th, 97.5th) percentiles of PPV and NPV in the simulation were 61% (29%, 85%) and 99% (97%, 99%), respectively, and within the expected ranges given the validation data. The wider quantiles, as compared to the validation substudy, reflects the incorporation of random error in the simulation. Based on this, our quantitative bias analysis suggested that the true prevalence of HCV in the risk-based cohort was 2.6% (95% simulation interval [SI]: 1.3%, 4.8%). For comparison, the naïve estimate shown in Table 1 was 1.2%, and was likely underestimated by several percentage points.

### 6.2. Differential Outcome Misclassification

To demonstrate differential misclassification, we considered the cohort characteristic race (operationalized as White versus non-White) as an exposure of interest, *Z*. The size of the validation subcohort was increased 100-fold to ensure sufficient cell counts in the stratifications. Our goal in this analysis was to estimate the RR of non-White race (relative to White race) as a predictor of true prevalence of HCV in the risk-based cohort. As before, we apply the algorithm from section 3.1 to construct the model of true prevalence based on the observed HCV EHR diagnosis and conditioned race. Working through Equations 3 and 4 yields the following estimates of the accuracy of our classifier in the subcohort by race (0 = White, 1 = Non-White; denoted in subscript): PPV_0_ = 38% (95% CI: 36%, 40%), PPV_1_ = 66% (95% CI: 65%, 67%), NPV_0_ = 98% (95% CI: 97%, 98%), NPV_1_ = 99% (95% CI: 99%, 99%), SN_0_ = 74% (95% CI: 71%, 77%), SN_1_ = 93% (95% CI: 92%, 94%), SP_0_ = 90% (95% CI: 90%, 91%), SP_1_ = 92% (95% CI: 92%, 92%). Table 3 presents the cross-tabulation of the validation subcohort stratified by race. We again simulated 1,000 potential sets of values of $\dot{X}$ for each individual, obtained for each iteration, and calculated the 2.5, 50, and 97.5 percentiles of the resultant simulated distribution of RR that accounts for misclassification and sampling errors.

**Figure 4.**
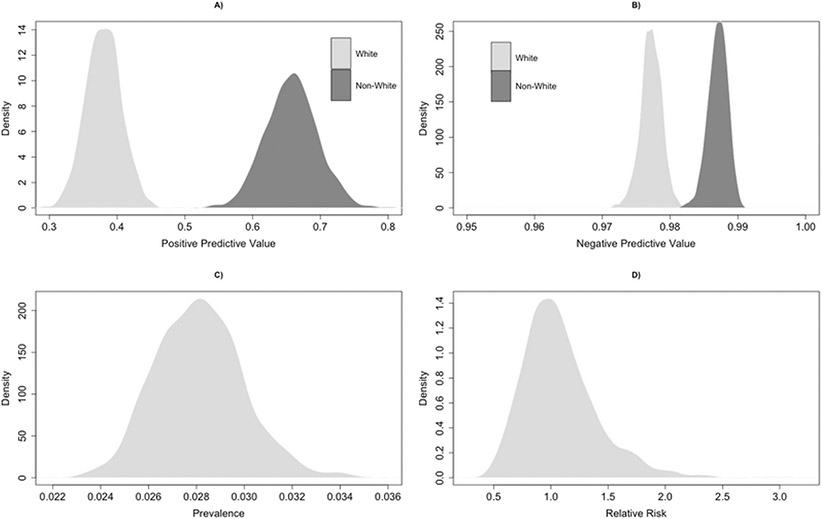
Density plots depicting the distribution of simulated positive predictive value (A), negative predictive value (B), true prevalence (C), and relative risk (D) obtained during the quantitative bias analysis of differential outcome misclassification.

The distribution of select parameters realized during this simulation are shown in Figure 4. The median (2.5th, 97.5th) percentiles of PPV_0_, PPV_1_, NPV_0_, and NPV_1_ in the simulation were 38% (33%, 42%), 66% (59%, 73%), 98% (97%, 98%), and 99% (98%, 99%), respectively, and within the expected ranges given the validation data. The wider quantiles, as compared to the validation substudy, again reflect the incorporation of random error in the simulation, as well as the smaller sample size (recall the size of the validation substudy for differential misclassification was increased 100-fold).

Using a modified Poisson regression with robust standard errors (Zou, 2004), we estimated a naïve RR in the risk-based cohort of the association of race with an EHR diagnosis of HCV. The naïve analysis suggested that patients of non-White race were 1.73 times as likely of having a diagnosis of HCV in the EHR compared to White race (RR = 1.73, 95% CI: 1.00, 2.98). Our quantitative bias analysis suggested that the median of non-White race versus White race of true HCV infection was 1.03 (95% SI: 0.59, 1.86). In other words, the differential misclassification present in the risk-based cohort biased the results away from the null and failure to consider this misclassification may have resulted in incorrect inference on the association of race with HCV in these data.

To account for the possibility of implausible values arising during the bias analysis simulation, we retained the likelihood of the models estimated in step 4 of the differential misclassification algorithm in section 3.1. The distribution of these likelihoods was approximately normal, and the weighted median was 1.04 (95% SI: 0.56, 1.97). The agreement between the weighted and unweighted results provided reassurance that our bias analysis was not susceptible to implausible combinations data and errors.

A summary of all accuracy measures of the EHR HCV classifier may be found in Table 4. The analytic code in R used in the case study is available to download from https://doi.org/10.5281/zenodo.5899411.

## 7. Discussion

### 7.1. Comments on the Case Study

In the validation study, we observed that in a risk factor-based screening model with low overall seroprevalence of HCV, absence of a diagnostic code is a strong indicator of lack of HCV, whereas presence of a diagnostic code translates to chronic HCV infection only about half the time. There are multiple reasons for this: individuals may have been antibody positive but PCR negative (e.g., treated and cured or have spontaneously cleared infection) or may have been misdocumented in the EHR (e.g., based on erroneous self-report or provider/documentation error). Therefore, when recalling patients from the EHR who are documented as HCV positive, confirmatory testing is warranted to confirm active infection.

The prevalence of chronic HCV in the risk-based cohort was observed to be 1% (an underestimation due to misclassification), the prevalence in the universal cohort among individuals with no indication of HCV in EHR was 2%, and the overall prevalence at this FQHC may even be higher as our quantitative bias analysis suggested. This indicates that a screening strategy that relies on known or disclosed risk factors will miss individuals who have HCV. Individuals may not disclose risk factors, if potentially stigmatizing, or otherwise be unaware that such risk factors exist. As such, the current push in the United States is for universal HCV screening regardless of known (or unknown) risk factors (Saab et al., 2018). Indeed, the U.S. Preventative Services Task Force now recommends that all individuals aged 18–79 years be screened at least once for HCV (USPSTF et al., 2020). Nevertheless, the modest uptake of HCV testing in our universal cohort (24%) suggests the challenges that lay ahead to convince otherwise asymptomatic individuals to submit to this test in a primary care setting of an FQHC.

Our work has implications for understanding the potential impact of bias in studies of HCV. When an individual’s true status is unknown, researchers must be aware of the possibility of misclassification, as well as the hypothesized HCV prevalence if working with predictive values. Seroprevalence (antibody positive) of HCV in a general medical setting, such as our FQHC, is likely to be substantially lower than in a high-risk setting, such as a syringe exchange program. One such syringe exchange program observed a seroprevalence of approximately 70%; people living with HIV who also inject drugs may have even higher HCV rates (Salek et al., 2017). Consequently, PPV and NPV observed in our sample, being dependent on prevalence, may not apply in higher-risk settings. However, SN and SP are independent of prevalence and therefore our estimates of these misclassification parameters may be useful in sensitivity analyses to assess the degree of information bias across many settings where the testing regime is like the one that we evaluated. Our bias analysis also suggested that apparent excess of HCV risk by race in this FQHC may have been due to differential misclassification, namely, greater undercounting of cases among White patients. This could conceivably happen if individuals who were non-White were more likely to be vetted for HCV, as there is no plausible biological difference in infection by race. Normally, we would not expect SN of 'testing' to vary by race, but in this case study, as noted in section 5, the only 'test' result in the earlier cohort was the presence of a record in the EHR. The presence or absence of this kind of record could be influenced by physician perceptions much more readily than any laboratory result.

There are several limitations to this study. First, in the estimation of the PPV, only individuals with documented, confirmed HCV received additional clinical workup. Thus, validation results are specific to risk-based screening programs, and not necessarily transportable to other HCV screening models. Second, EHR documentation practices may vary dependent on the clinical setting. While this study provides estimation from a single urban FQHC, the generalizability to other high-risk health center populations should be evaluated on a case-by-case basis. Third, lab results in both cohorts were only available if a patient fulfilled the order and there are likely factors related to patients’ health-seeking behaviors not captured, which may also relate to their HCV status. For example, if we underestimated prevalence, our PPV will be too low and our NPV will be too high. Fourth and finally, by combining patients from the universal and risk-based cohorts, we assumed exchangeability of the patient population at different periods of times. Reassuringly, we did not observe a material difference in the characteristics between those who fulfilled a lab order and those who did not, nor between the two cohorts under study. Strengths of our work included the use of multiple indicators of HCV, a large contemporary cohort, and patient outreach.

If we did not have access to a validation subcohort in this study, we still could have proceeded with a bias analysis. This would have required an alternate source for the parameters used in section 6. There are numerous published articles on the accuracy of ICD codes in the EHR (see, for example Goff et al., 2012; Schneeweiss & Avorn, 2005). Even if we could not find the exact diagnostic codes used in our study, we may have posited that the process of misdiagnosis was similar but decreased the precision of the bias correction parameters to acknowledge the greater uncertainty. Relatedly, operationalizing a clinical phenotype based on an ICD code alone is potentially problematic and in practice it is necessary to consider multiple criteria (Richesson et al., 2021). Indeed, we observed this during our validation of true HCV infection via manual chart review, where presence of a diagnostic code alone was insufficient to fully determine someone’s HCV status. Potentially, this could be automated via natural language processing of free-text clinical notes, an emerging area in EHR research (Juhn & Liu, 2020), but any residual errors from such automation may need to be again accounted for in quantitative bias analysis. To this effect and for some problems, it may be essential to define the gold standard of diagnosis, such as using a panel of independent physicians who review medical records and reach a consensus on the diagnosis.

In short, our validation study quantified the potential for misclassification of HCV diagnosis in the EHR as well as an underestimation in active HCV in a risk-based screening model. In the research setting, which often relies on imperfect data, investigators can perform a quantitative bias analysis using our misclassification parameters. Our findings further underscore the need for universal HCV screening in clinical practice.

### 7.2. Concluding Remarks

As we have detailed in this article, the secondary analysis of EHR health outcome data requires a careful evaluation of the quality of the data handed to data scientists. Certainly, the use of EHR data for research purposes will only be increasing, as more healthcare practices continue to adopt their use, both inpatient and outpatient (Adler-Milstein et al., 2017; Office of the National Coordinator for Health Information Technology, 2021). Thus, the need for measuring and evaluating the accuracy of these data will continue. Ideally, if the EHR data are complete and accurate, one would not need to employ the methodological tools we have described herein. Although this ideal may not be attainable (there may always be measurement error based on imperfect diagnostic tools and procedures), by engaging in interdisciplinary research, including forming teams comprising those who capture and enter the data into EHRs (i.e., clinicians and ancillary staff) as well as those who design and deploy the EHRs in the health care setting (i.e., informaticians), we can minimize our reliance upon tenuous assumptions at the research stage. Despite our focus on outcome misclassification, there are other threats to validity that arise in EHR research. Gianfrancesco & Goldstein (2021) articulate four central challenges to validity of EHR-based research: issues of selection and representativeness, data availability and interpretation (including measurement error), missing measurements, and missing visits. No EHR study would be complete without reflecting upon all such considerations, and one can conduct a sensitivity analysis of the total bias that includes multiple threats to validity concurrently (Smith et al., 2021).

The methods we presented should place minimal burden on researchers beyond the typically massive effort involved in extraction and cleaning of EHR-derived data, and there are multiple approaches for validation and quantitative bias analysis of misclassified data (see, for example, Lash et al., 2009). However, we recognize that it may not always be feasible to conduct a validation study for a number of reasons, including lack of time, resources, or ability to perform primary data collection on a sample of patients. In such cases, a literature review may yield the parameters necessary to conduct a quantitative bias analysis. Yet even a comprehensive review may come up short: the researcher should not throw their hands in the air and conclude there is nothing to be done. One can always turn to expert opinion or informed guesses about where these parameters may he, and then conduct a bias analysis. As such, quantitative bias analysis is an extremely flexible approach. In fact, quantitative bias analyses extend beyond the bounds of data accuracy: similar approaches can handle cases of selection bias and residual confounding (again, see Lash et al., 2009, and Smith et al., 2021). Beyond the case study demonstrated in this article, we refer readers to these other examples of quantitative bias analysis applied to misclassified outcome data (Bodnar et al., 2010; Burstyn et al., 2020; Goldstein et al., 2016; Goldstein et al., 2021; Jurek & Maldonado, 2016; Srugo et al., 2021; Wesselink et al., 2018).

To expound one such example, in Bodnar et al., the authors assessed the relationship between prepregnancy body mass index (BMI) and several adverse pregnancy outcomes. The authors posited that the self-reported weight captured in the EHR was inaccurate and thus the calculated BMI categories were potentially misclassified. As the authors lacked an internal validation study, they turned to the National Health and Nutrition Examination Survey (NHANES), a representative health survey of nonincarcerated adults in the United States. NHANES captured both self-reported weight and measured weight among women of childbearing age, and therefore the authors were able to estimate measures of the accuracy of self-reported weight (in this case, PPV and NPV). After conducting a probabilistic bias analysis, they observed an attenuation of the BMI effect, indicating the misclassified estimates were biased away from the null. This otherwise could not have been anticipated given the polytomous exposure.

In differential misclassification situations, a question arises whether misclassification parameters are correlated among strata. There is a paucity of empirical evidence, but intuition has led some (e.g., Lash et al., 2009) to assert that such a correlation, namely, positive correlation of SN in exposed and unexposed, positive correlation of SP in exposed and unexposed, is the only sensible default. The intuitive rationale is that we would update our belief about SN in one group within a study if SN in another group was revealed; that is, there is something common among all mechanisms by which errors arise in one study (or for a given measurement instrument, process) (Gustafson & McCandless, 2014). We do not explicitly address this issue, but insofar as coefficients in multiple validation regression models of (*X*∣ *W,Z*) are interdependent, our approach perhaps captures some of this dependence. The matter deserves a more in-depth look and ultimately may lead down the path of elucidation of multivariate priors on misclassification parameters with the correlation explicitly specified. Estimate of prevalence under differential misclassification, when covariate *Z* is considered, is not straightforward, because distribution of *Z* must be taken into account. In such a setting, procedures that estimate marginal predicted probability can be inserted into our algorithm (Muller & MacLehose, 2014).

In conclusion, data science has a well-earned reputation of focusing on computational solutions to complex problems. Certainly, EHR data qualify as the latter. Validation studies combined with quantitative bias analysis satisfy the former.

## Figures and Tables

**Table 1.** Characteristics of the Two Cohorts of Patients Seen at an Urban Federally Qualified Health Center for Classifying True Chronic Hepatitis C Virus Infection Status Based on an Electronic Health Record Diagnosis

	Risk-Based Cohort	Universal Cohort		
Characteristic	Total (*n* = 3,773)	Total (*n* = 1,445)	Fulfilled lab order (*n* = 341, 24%)	Did not fulfill lab order (*n* = 1,104, 74%)
Age in years, median (IQR)	38 (29–51)	43 (31–56)	43 (31–56)	43 (30–56)
Sex, *n* (%)				
Female	2,540 (67%)	968 (67%)	219 (64%)	749 (68%)
Male	1,233 (33%)	477 (33%)	122 (36%)	355 (32%)
Race, *n* (%)				
White	1,161 (31%)	436 (30%)	93 (28%)	343 (31%)
Non-White	2,564 (69%)	994 (70%)	243 (72%)	751 (69%)
Ethnicity, *n* (%)				
Non-Hispanic	2,828 (75%)	1,104 (77%)	262 (77%)	842 (77%)
Hispanic	934 (25%)	335 (23%)	79 (23%)	256 (33%)
Insurance, *n* (%)				
Private	919 (32%)	409 (36%)	95 (34%)	314 (36%)
Nonprivate	1,957 (68%)	738 (64%)	181 (66%)	557 (64%)
Chronic Hepatitis C virus infection status, *n* (%)				
ICD code in EHR	77 (2%)	0 (0%)	0 (0%)	0 (0%)
Confirmed^†^	47 (1%)	--^‡^	5 (2%)	--^‡^

*Note.* IQR = interquartile range; ICD = International Classification of Diseases; EHR = electronic health record.

† Those with a positive confirmatory RNA test or documented HCV treatment.

‡ Unable to estimate due to missing data.

**Table 2.** Contingency Table of an EHR Indicator of HCV by True HCV Status in the Validation Subcohort

	True HCV status (*X*)				
**EHR diagnosis (*W*)**	Present (*X*=1)	*n*	Absent (*X* = 0)	*n*	Totals
Present (*W* = 1)	True positive	a = 47	False positive	b = 30	a + b = 77
Absent (*W* = 0	False negative	c = 5	True negative	d = 336	c + d = 341
Totals		a + c = 52		b + d = 366	a + b + c + d = 418

*Note.* HCV = chronic hepatitis C virus infection; EHR = electronic health record; Sensitivity = a / (a + c); Specificity = d / (b + d); Positive predictive value = a / (a + b); Negative predictive value = d / (c + d); Prevalence = (a + b) / (a + b + c + d)

**Table 3.** Contingency Table of an EHR Indicator of HCV by True HCV Status, Stratified by Race, in the Validation Subcohort^†^

White (*Z* = 0)	True HCV Status (*X*)				
**EHR diagnosis (*W*)**	Present (*X* = 1)	*n*	Absent (*X* = 0)	*n*	*Totals*
Present (*W* =1)	True positive	a = 613	False positive	b = 1,015	a + b = 1,628
Absent (*W* = 0	False negative	c = 192	True negative	d = 9,105	c + d = 9,297
*Totals*		a + c = 805		b + d = 10,120	a + b + c + d = 10,925
Non-White (*Z* = 1)	True HCV status (*X*)				
**EHR diagnosis (*W*)**	Present (*X* = 1)	*n*	Absent (*X* = 0)	*n*	*Totals*
Present (*W* = 1)	True positive	a = 4,173	False positive	b = 1,979	a + b = 6,152
Absent (*W* = 0)	False negative	c = 307	True negative	d = 23,909	c + d = 24,216
*Totals*		a + c = 4,480		b + d = 25,888	a + b + c + d = 30,368

*Note.* HCV = chronic hepatitis C virus infection; EHR = electronic health record; Sensitivity = a / (a + c); Specificity = d / (b + d); Positive predictive value = a / (a + b); Negative predictive value = d / (c + d); Prevalence = (a + b) / (a + b + c + d).

† The size of the validation subcohort was increased 100-fold to ensure sufficient cell counts in the stratifications.

**Table 4.** Summary of accuracy measures of the EHR indicator of HCV obtained from the validation sub-cohort, nondifferential and differential with respect to race.

	Estimate (95% confidence interval)		
Parameter	Nondifferential	Differential by Race	
		*White*	*Non-White*
Sensitivity	90% (82%, 98%)	76% (71%, 77%)	93% (92%, 94%)
Specificity	92% (89%, 95%)	90% (90%, 91%)	92% (92%, 92%)
Positive Predictive Value	61% (51%, 73%)	38% (36%, 40%)	66% (65%, 67%)
Negative Predictive Value	99% (97%, 100%)	98% (97%, 98%)	99% (99%, 99%)

*Note.* HCV, chronic hepatitis C virus infection; EHR, electronic health record.
